# A review of use of external data and update on reporting standards in Sequential Multiple-Assignment Randomised Trials

**DOI:** 10.1177/17407745251385535

**Published:** 2025-11-05

**Authors:** Isaac J Egesa, Laura Bonnett, Richard Emsley, Anthony Marson, Catrin T Smith

**Affiliations:** 1 Health Data Science, Institute of Population Health, University of Liverpool, Liverpool, UK; 2 Institute of Psychiatry, Psychology & Neuroscience, King’s College London, London, UK; 3 Institute of Systems, Molecular and Integrative Biology, University of Liverpool, Liverpool, UK; 4 The Walton Centre NHS Foundation Trust, Liverpool, UK

**Keywords:** Sequential Multiple-Assignment Randomised Trial, external data, routine data, adaptive interventions, systematic review

## Abstract

**Background:**

The Sequential Multiple-Assignment Randomised Trial (SMART) design is considered the gold standard for developing adaptive interventions, which tailor treatments to individual patient characteristics and responses. While SMART offers a rigorous framework aligned with real-world clinical decision-making, it is often complex, time-consuming, and costly. As interest in SMART design grows, there is increasing recognition for the need to improve its implementation through more explicit guidance and best practices. Efficiency gains may also be possible by incorporating external data to inform their design, conduct, and analysis. This review aimed to identify all published trials using the SMART design, summarise their design, conduct, and reporting practices and evaluate the use of external data in their implementation.

**Methods:**

We searched PubMed, Medline, PsycINFO, Scopus, and Web of Science databases for all SMART up to June 30, 2024. External data were defined as non-simulated individual patient data collected outside the main SMART to supplement or inform the main trial.

**Results:**

We included 80 SMART, of which 35 (44%) were completed and 45 (56%) were ongoing. Most trials reported two phases of randomisation (93%), with the primary aim focusing on evaluating main effects (81%) of interventions at the first stage of randomisation. There was inadequate reporting of several key aspects, including sample size estimation, statistical analysis software, allocation concealment, data missingness, multiple testing, sensitivity analysis, and the use of SMART in the title. Seventeen (21%) SMART (4-completed trials and 13-trial protocols) referred to the use of external data from electronic health records (n = 12) and registries (n = 5). External data was used for recruitment (n = 11), outcome measures (n = 6), and to provide baseline covariate information (n = 1).

**Conclusion:**

SMART designs are increasingly used to develop adaptive interventions across diverse clinical contexts, yet key methodological features and basic components remain inconsistently reported. This limits transparency, reproducibility, and potential for translation into routine care. Although external data are widely used in standard randomised controlled trials, their use in the SMART is still limited, likely due to methodological and infrastructural challenges and the absence of tailored reporting standards. To improve the efficiency and generalisability of SMART designs, expert-led extensions of CONSORT and SPIRIT guidelines are needed, including specific recommendations for reporting external data use. Future research should explore optimal external data sources for informing SMART components and promote interdisciplinary collaboration and training to support high-quality implementation.

## Introduction

Adaptive interventions, also known as personalised medicine, describe the principle of tailoring treatments to individual patient characteristics and response to optimise care.^1^ This is formalised as a sequence of decision rules, at given decision points, that establishes treatments for patients, or the next course of action, based on up-to-date information. Adaptive interventions, therefore, reflect the ongoing multi-stage decision-making by clinicians in practice.

Finding the best adaptive interventions would be a way to apply evidence-based personalised medicine in the real world.^2^ To develop and refine adaptive interventions, an experimental design known as the Sequential Multiple Assignment Randomised Trial (SMART) was proposed.^3^ SMART re-randomises participants at multiple stages based on pre-specified rules.^3^ Adaptive interventions are embedded in a SMART. Patients are randomised to initial and subsequent treatments based on treatment response and individual characteristics. Each randomisation stage in SMART corresponds to a critical decision point in adaptive interventions. This enables researchers to build or refine adaptive interventions within a SMART, making it a robust tool for personalised medicine.

Although SMART designs provide a rigorous framework for developing adaptive interventions in chronic diseases and other health-related conditions,^4^ their implementation continues to encounter methodological and practical challenges, including limited expertise and their inherent complexity.^5–11^ To mitigate these challenges, SMART studies must be carefully designed with clear objectives. Like standard trials, their design, conduct, and reporting are guided by the CONSORT statement and International Conference on Harmonisation E9.^12,13^ Systematic reviews of SMART design, conduct, and reporting are crucial for closing the knowledge gap, improving transparency and interpretability, informing guidelines, promoting best practices, and enhancing utilisation. Recent reviews by Bigirumurame et al.^7^ and Lorenzoni et al.^8^ highlighted the need for transparency and comprehensive reporting in SMART. With the growing interest in personalised medicine, SMART designs are likely to gain a firm foothold in clinical trials to aid in developing adaptive interventions,^11^ thus necessitating their continuous updates in design, conduct, and reporting.

Despite their alignment with real-world clinical practice,^3^ SMART designs can be time-consuming and costly. These burdens may be reduced by using external data to inform key aspects of their design. External data – defined as individual patient data collected outside the main trial – is increasingly used to support randomised control trial’s (RCT) design and conduct.^14,15^ External data sources like electronic health records (EHRs), including Clinical Practice Research Datalink,^16^ and disease registries can guide SMART’s planning and implementation.^17^ Specifically, they can help define decision rules and decision point(s),^18^ identify at-risk populations (tailoring variables), assess feasibility, and support sample size estimation.^19,20^ The growing availability of EHRs and registries has led regulators and industry to recognise their value in practice and drug development,^21,22^ as reflected in the CONSORT extension for reporting external data in RCTs^23^ and the ongoing development of the SPIRIT guidelines.^24^ Establishing similar standards for SMART designs is crucial to improve transparency and reproducibility.

Herein, we present a review that evaluates the design, conduct, and reporting of SMART studies and assesses their use of external data in published literature.

## Methods

The review protocol is available on the Open Science Framework.^25^

### Search strategy

We expanded the search terms from Bigirumurame et al.^7^ to include additional SMART-related terms (Supplemental Appendix S1). A focused search was conducted in Scopus, Medline (Ovid), Web of Science, PubMed and PsycINFO for articles published between 2020 and June 30, 2024. The 2020 start date was chosen to build on Bigirumurame et al’.s^7^ prior review, which covered literature up to 2019, to ensure continuity and avoid duplication. We also screened reference lists and eligible articles from their full-text review.

### Eligibility criteria

We included all interventional studies using a SMART design, published in English, with full-text reports. SMART design was defined as involving ≥ 2 stages of randomisation based on pre-defined rules.^3^ External data was defined as non-simulated individual patient data collected outside the main SMART study to supplement or inform the trial. We excluded book chapters, dissertations, commentaries, editorials, mixed-methods articles, systematic reviews, conference papers, and feasibility studies.

### Article screening

All articles were imported into EndNoteX20^26^ and duplicates removed. Two reviewers (IE and CTS) independently screened titles, abstracts, and keywords using Rayyan.^27^ For full-text screening, both reviewers assessed all articles independently, noting reasons for exclusion. Discrepancies were resolved by consensus following discussion between reviewers.

### Data extraction

Data were extracted using a standardised Microsoft Excel spreadsheet,^28^ piloted on 12 completed trials from Bigirumurame et al.^7^ Extracted data covered study characteristics, SMART design and conduct, aims, analysis, reporting, and external data use. The first reviewer (IE) extracted all data, which was cross-verified by an independent reviewer (CTS).

### Data analysis

All analyses were performed in R version 4.3.0^29^ using descriptive statistics to report frequencies and proportions of study characteristics and trial types.

### Quality assessment

Quality assessment was not performed as the focus was on SMART study design, conduct, reporting, and use of external data.

## Results

### Search results

**Figure 1.**
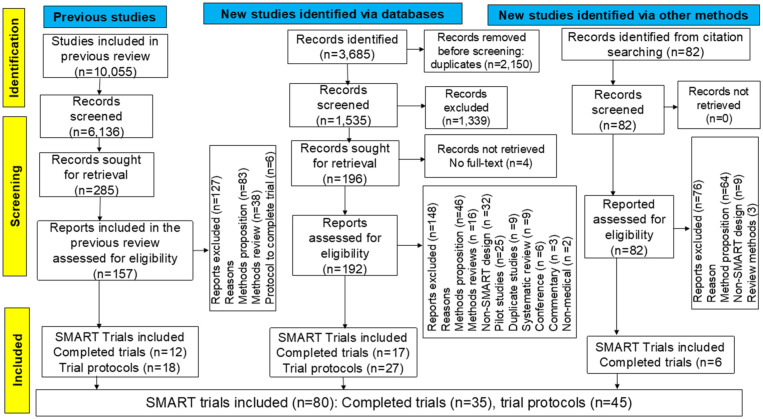
PRISMA flowchart.

Figure 1 presents the PRISMA flowchart. Of the 3685 records identified, 2150 duplicates were removed, leaving 1535 for title and abstract screening. Backward citation searching added 82 records. After excluding 1339 records, 192 full-text articles from the updated search and 82 from the citation search were assessed. Four records lacking full-texts were excluded. In total, 431 full-text articles were screened, including 274 from the updated search and 157 from the previous review.^7^ Unlike the earlier review,^7^ we excluded reviews and methodological papers to focus on randomised trials. The final sample included 80 independent SMART; 35 (44.0%) completed trials^30–64^ and 45 (56.0%) protocols.^65–109^

### SMART study characteristics

**Figure 2.**
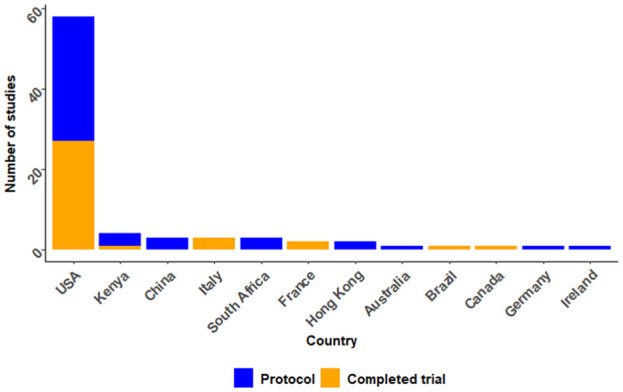
Included study publications by countries.

**Figure 3.**
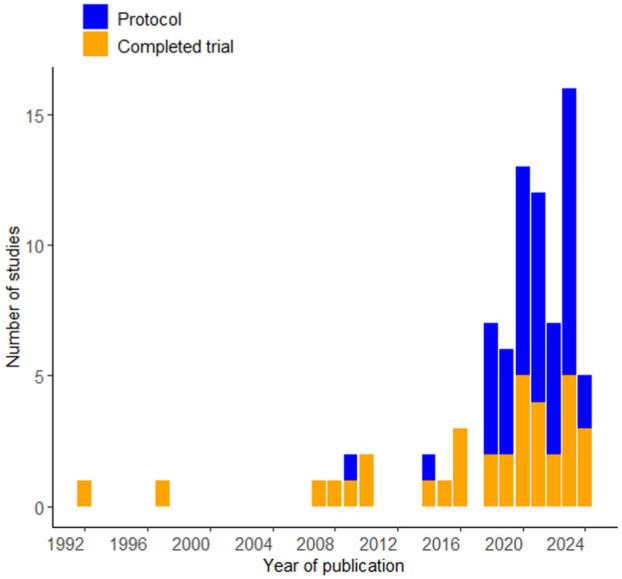
SMART studies published over the years.

Table 1 summarises characteristics of the 80 SMART. Studies were conducted in 12 countries, with the majority published in the United States (n = 58, 72.5%) (Figure 2). Studies were published between 1992 and 2024, with a notable increase in the number of studies published after 2018. Since the 2020 review,^7^ completed SMART nearly tripled and protocols doubled (Figure 3).

**Table 1.** Characteristics of SMART.

Characteristics	Completed trials (n = 35, %)	Trial protocols (n = 45, %)	Overall (N = 80, %)
Trial characteristics			
Field of health research			
Cancer	10 (28.6)	1(2.2)	11 (13.8)
Infectious diseases	1 (2.9)	9 (20.0)	10 (12.5)
Pain and physical health disorder	5 (14.3)	8 (17.8)	13 (16.3)
Neurological disorder	1 (2.9)	3 (6.7)	4 (5.0)
Psychiatric disorder	10 (28.6)	17 (37.8)	27 (33.8)
Substance use disorder	8 (22.9)	7 (15.6)	15 (18.8)
Population			
Children and adolescents	7 (20.0)	10 (22.2)	17 (21.3)
Adults	27 (77.1)	31 (68.9)	58 (72.5)
All age	1 (2.9)	4 (8.9)	5 (6.3)
Study setting			
Hospital	20 (57.1)	20 (44.4)	40 (50.0)
Community	9 (25.7)	15 (33.3)	24 (30.0)
Hospital and community	6 (17.1)	10 (22.2)	16 (20.0)
Funding			
Private	2 (5.7)	3 (6.7)	5 (6.3)
Public	23 (65.7)	41 (91.1)	64 (80.0)
Private and public	6 (17.1)	1 (2.2)	7 (8.8)
No funding	1 (2.9)		1 (1.3)
No information	3 (8.6)		3 (3.8)
NIH funding	18 (51.4)	31 (68.9)	49 (61.2)
Multicentre trial			
No	13 (37.1)	21 (46.7)	34 (42.5)
Yes	22 (62.9)	24 (53.3)	46 (57.5)
Reporting			
Study title identified as SMART			
No	16 (45.7)	7 (15.6)	23 (28.8)
Yes	19 (54.3)	38 (84.4)	57 (71.2)
Trial registration details			
No	5 (14.3)	2 (4.4)	7 (8.8)
Yes	30 (85.7)	43 (95.6)	73 (91.2)
Allocation concealment reporting			
No	17 (48.6)	15 (33.3)	32 (40.0)
Yes	18 (51.4)	30 (66.7)	48 (60.0)
Sample size estimation reporting			
No	7 (20.0)		7 (8.8)
Yes	28 (80.0)	45 (100)	73 (91.2)
Reported software for sample size estimation			
No	33 (94.3)	35 (77.8)	68 (85.0)
Yes	2 (5.71)	10 (22.2)	12 (15.0)
Reported missingness			
No	19 (54.3)	21 (46.7)	40 (50.0)
Yes	16 (45.7)	24 (53.3)	40 (50.0)
Pilot/feasibility study informing trial reported			
No	1 (2.9)	11 (24.4)	12 (15.0)
Yes	34 (97.1)	34 (75.6)	68 (85.0)
Blinding reported			
No	5 (14.3)	1 (2.2)	6 (7.5)
Yes	30 (85.7)	44 (98.8)	74 (92.5)
Analysis software reported			
No	20 (57.1)	30 (66.7)	50 (62.5)
Yes	15 (42.9)	15 (33.3)	30 (37.5)
Design and conduct			
Randomisation phases			
Two	33 (94.3)	41 (91.1)	74 (92.5)
Three	3 (2.9)	4 (8.9)	5 (6.3)
Four	1 (2.8)		1 (1.2)
Sample size			
Median (IQR)	360 (160-491)	400 (280-800)	377 (194-671)
Minimum-maximum	61-2876	36-6000	36-6000
Attrition rate, median % (IQR)			
First stage	11.3 (6.2-14.8)		
Second stage	17.5 (13.0-30)		
SMART model			
Prototypical	24 (68.6)	38 (84.4)	62 (77.5)
Responsive intervention	7 (20.0)	1 (2.2)	8 (10.0)
Unrestricted SMART	4 (11.4)	6 (13.3)	10 (12.5)
Study duration in months, median (IQR)	12 (6, 24)		
Number of initial treatments			
1	5 (14.3)	7 (15.6)	12 (15.0)
2	24 (68.6)	34 (75.6)	58 (72.5)
3	4 (11.4)	3 (6.7)	7 (8.8)
4	1 (2.9)		1 (1.3)
5	1 (15.6)	1 (2.2)	2 (2.5)
SMART aims considerations			
Randomisation stage of the primary aim			
First stage	29 (82.9)	28 (62.2)	57 (71.2)
Second stage	2 (5.7)	3 (6.67)	5 (6.3)
Both	4 (11.4)	14 (31.1)	18 (22.5)
Embedded adaptive interventions comparisons			
No	17 (48.6)	17 (37.8)	34 (42.5)
Yes	18 (51.4)	28 (62.2)	46 (57.5)
Selecting deeply embedded adaptive interventions			
No	32 (91.4)	27 (60.0)	59 (73.8)
Yes	3 (8.6)	18 (40.0)	21 (26.2)
SMART aim considerations			
Single aim	21 (60.0)	14 (31.1)	35 (43.7)
Two aims	13 (37.1)	22 (48.9)	35 (43.7)
Three aims	1 (2.9)	9 (20.0)	10 (12.5)
Main effect aim consideration as the primary aim			
No	2 (5.7)	13 (28.9)	15 (18.8)
Yes	33 (94.3)	32 (71.1)	65 (81.2)
Embedded adaptive intervention consideration as the primary aim			
No	32 (91.4)	34 (75.6)	66 (82.5)
Yes	3 (8.6)	11 (24.4)	14 (17.5)
Optimisation aims consideration as the primary aim			
No	34 (97.1)	43 (95.6)	78 (97.5)
Yes	1 (2.9)	2 (4.4)	3 (2.5)
Analysis considerations			
Use of multiple testing			
No	31 (88.6)	35 (77.8)	66 (82.5)
Yes	4 (11.4)	10 (22.2)	14 (17.5)
Analysis method for primary aim			
ANOVA	1 (2.9)		
Generalised estimating equation	3 (8.6)		
Generalised linear model	12 (34.3)		
Linear mixed-effect model	7 (20.0)		
T-test	1 (2.9)		
Survival	11 (31.4)		
Analysis type			
Intention to treat analysis	29 (82.9)	34 (75.6)	63 (78.7)
Not specified	6 (17.1)	11 (24.4)	17 (21.3)

SMART: Sequential Multiple-Assignment Randomised Trial; IQR: interquartile range; ANOVA: analysis of variance.

Psychiatric disorders were most studied (n = 27, 33.8%), followed by substance use disorders (n = 15, 18.8%), pain/physical disorders (n = 13, 16.3%), cancer (n = 11, 13.8%), and infectious diseases (n = 10, 12.5%) and neurological disorders (n = 4, 5.0%). The psychiatric disorders reported included depression,^58,59,66,67,74,84,85,88^ anxiety,^59,96^ post-traumatic stress disorder,^33,88,100,101^ attention-deficit hyperactivity disorder,^50,69^ sleep disorders,^47,74,76^ suicide,^90,93^ schizophrenia,^61,89,92^ bipolar disorder,^36^ obsessive-compulsive disorder,^32^ eating disorder,^38^ autism,^41^ and intimate partner violence.^99^ Pain/physical disorders included chronic pain,^34,60,78,80,82^ weight loss/obesity or diabetes,^56,79,81,86^ osteoarthritis,^40^ and burns.^83^ Infectious diseases were mainly HIV/AIDS,^37,65,70,73,91,102,103^ malaria,^108^ and COVID-19.^98,106^ Neurological disorders included dementia,^87,105^ stroke,^72^ and learning disability.^42^ Substance use disorders include alcohol use disorder,^45,46,49,94,104^ smoking,^31,35,54,75,107^ and cocaine and opioids.^52,55,95,97^

Most trials focused on adults (≥18 years) (n = 58, 72.5%), followed by children/adolescents (n = 17, 21.3%), and all-age groups (n = 6, 6.3%). Half of the trials used hospital/clinic samples; about one-fifth combined community and hospital samples, and one-third sampled from the community alone. Most studies were funded by public funding (n = 71, 88.7%), with the National Institute of Health (NIH) being the main funder (61.3%) for publicly funded studies.^65,67,68,70,73–75,79,97,100,105,109^

### Design characteristics of SMART

Almost all trials (n = 74, 92.5%) reported two randomisation phases. In the first randomisation stage, 58 (72.5%) randomised between two interventions, seven between three, one between four, and two between five. Twelve studies (15.0%) used a run-in intervention model, where participants received the same treatment before first randomisation.^34,45,46,54,58,68,82,84,85,91,92,97^ Over three-quarters of trials (n = 62, 77.5%) were prototypical SMART, where re-randomisation occurs for non-responders of stage 1 interventions. Ten trials (12.5%) were unrestricted SMART (re-randomisation occurs regardless of response status), and eight (10.0%) were responsive SMART (re-randomisation occurs to responders to initial treatment). Most trials (n = 74, 92.5%) randomised at individual level, while six (7.5%) randomised at the cluster level. The median sample for completed trials was 360 participants (interquartile range (IQR), 160–491), with the smallest and largest trial recruiting 61 and 2876 participants, respectively. Follow-up duration ranged from 2 days (in an emergency setting) to 96 months, with a median of 12 months (IQR 6–24).

### Aims considerations in SMART

SMART design evaluates aims for main effect, embedded adaptive interventions, and optimisation. The main effect aims to identify the best initial intervention or effective option for responders/non-responders. Embedded adaptive intervention aims to compare or/and identify effective adaptive interventions, while the optimisation aim, often referred to as hypothesis generating, explores deeply tailored adaptive interventions.^6^ Trials considered all three aims in 10 (12.5%) and at least two in 35 (43.7%) trials. Over three-quarters (n = 65) of trials considered the main effect aim as the primary aim, while embedded adaptive intervention and optimisation were considered in 14 (17.5%) and 2 (2.5%), respectively. One trial considered the main effect and embedded adaptive intervention as the primary aim.^52^ Over two-thirds of trials (n = 57) focused on the primary aim in first-stage randomised interventions, five in second-stage, and 18 across all stages. Forty-six trials compared multiple embedded adaptive interventions, while 21 evaluated a single adaptive strategy that optimises outcomes.

### Analysis considerations in SMART

Completed trials used analysis of variance (ANOVA) (n = 1), t-test (n = 1), generalised estimation equation (GEE) (n = 3), generalised linear model (GLM) (n = 11), survival analysis (n = 11), and mixed effects model (n = 8) analytical techniques to address the primary research questions. Unlike completed trials, protocols described the analysis plans in a high-level summary, often mentioning broad methodological approaches in SMART without providing specific details. For example, protocols referenced potential analysis techniques in SMART,^83,95^ or mentioned specific techniques such as mixed-effect regression^76^ and longitudinal models.^82^ Only 22 (48.9%) protocols reported a detailed analysis plan for the primary research question, including GEE (n = 5), mixed effects model (n = 9), GLM (n = 6), marginal mean model (n = 1), and targeted maximum likelihood (n = 1). Q-learning was intended/used for secondary or exploratory analysis in 14 trials.^49,59,66,70,78,80,83,84,88,91,96,97,101,108^

Multiple comparisons of adaptive interventions in SMART require pre-specified or reported adjustments to control for Type 1 error.^110–112^ Only four completed trials reported doing so, using the Bonferroni method,^48,58^ Hochberg adjustment,^61^ or discussing the impact of multiple testing on effect estimate.^42^ Ten protocols intended to assess multiple testing in the analysis plan, including the use of a composite outcome strategy to avoid multiplicity.^77^

### Reporting of SMART

One-third (28.8%, n = 23) of the studies did not mention SMART design in the title, and about half of the completed trial reports omitted it. Trial registration was reported in 73 trials (91.2%), but only 35 trials (43.8%) reported the assignment model as sequential. Other assignment models reported included parallel (n = 26), factorial (n = 9), cross-over (n = 1), and single group (n = 1), while eight studies (10.0%) did not specify a model. SMART design features, such as tailoring variables, decision rules, and critical decision points, were exclusively reported in 17 (21.3%), 8 (10.0%), and 3 (3.8%) studies, respectively.

Seventy-four trials (92.5%) reported blinding status, which included: no blinding (n = 24), single (n = 35), double (n = 10), triple (n = 1) or quadruple blinding (n = 4). Allocation concealment was exclusively reported in 48 (60.0%) trials. Sample size estimation details were reported in 73 studies (91.2%), though software used was reported in 12 (15.0%), including SAS, G Power, PASS, R, PS, and STPLAN. Pilot or feasibility studies before the main SMART or development of the SMART protocol were reported in 12 (15.0%) studies. Statistical software was reported in 30 trials (37.5%), including East Software Cytel (n = 1), MPLUS (n = 1), SPLUS (n = 1), SPSS (n = 3), SPSS and SAS (n = 1), SAS and R (n = 3), SAS (n = 8), R (n = 10), STATA and R (n = 1), and STATA and SAS (n = 1). Missing data or plans to address it were reported in 40 trials (50.0%).

### External data in SMART

External data use was reported in 17 trials (21.2%); 4 completed trials^40,48,55,60^ and 13 protocols^65,67,68,70,73–75,79,97,98,100,105,109^ (Table 2). It was mostly reported in pain and physical disorder, infectious diseases and psychiatric disorders in four studies each (23.5%). Almost all the studies that used external data were publicly funded (94.1%), of which 75.0% were NIH funded. Most studies were from the United States (82.4%), involved multisite trials (58.8%) and were published beyond 2020 (70.5%). The majority used EHRs (n = 12) than registries (n = 5). Each study reported a single external data source. External data usage ranged from minimal to substantial across trials and protocols. All studies used external data to supplement other data sources, rather than relying exclusively on it for primary purposes (Table 2).

**Table 2.** External data in SMART.

	Completed trial (n = 35, %)	Trial protocols (n = 45, %)	Overall (N = 80, %)
Use of external data			
No	31(88.6)	32 (71.1)	63 (78.8)
Yes	4 (11.4)	13 (28.9)	17 (21.2)
External data type			
Registry	2 (5.7)	3 (6.7)	5 (6.3)
Electronic health record	2 (5.7)	10 (22.2)	12 (15.0)
Purpose of external data			
Recruitment	4 (11.4)	7 (15.6)	11 (13.8)
Outcome measure		6 (13.3)	6 (7.5)
Baseline covariate measure		1 (2.2)	1 (1.3)

### Completed trials

Of the four completed trials that used external data: two used data from EHRs and two used patient registries. Completed trials used external data to supplement recruitment or enrolment of participants. Medical records were reviewed alongside self-referral, direct provider referral, and community inquiries to identify potentially eligible participants in EHRs.^48,60^ For registries: a university research registry to supplement primary care in the recruitment of participants^40^ and a smoking registry to prompt potential participants on their willingness to participate in the trial or invite them to participate^54^ were used.

### Protocols

Thirteen SMART protocols used external data from EHRs (n = 10) and registries (n = 3). Seven studies planned using EHRs or registry to identify potential participants,^74,75,100,105,109^ enrol participants,^67^ and send invitation messages to potential participants.^79^ Six protocols intended to use external data to measure outcomes, including blood pressure, laboratory tests (glycated haemoglobin, HIV viral load, urinalysis and lipid profile), adherence, and suicide screening uptake evaluation.^65,68,70,73,97,109^ One protocol planned using immunisation status data from EHR as a baseline covariate information to analyse the study outcome.^98^ One protocol planned using data for both recruitment and outcome measure.^109^

## Discussion

This review identified 80 SMART (35 completed trials and 45 protocols), twice as many as in the previous review in a span of 4 years. Most trials were published in the United States, recruited adults from hospitals, conducted in multicentre settings, publicly funded, and focused on psychiatric disorders.

### SMART design features

Most SMART began with two treatment options and two phases of randomisation, consistent with prior studies.^7,8^ Although SMART designs can address three main scientific aims,^113^ only 12.5% of the trials explored all. This suboptimal use may reflect gaps in methodological expertise, complexity of multi-aim designs, funding constraints^6,20^ or the field’s early focus on narrow objectives. Encouragingly, training resources have expanded in recent years. Notable contributions include annual online SMART-workshops,^114^ the d3centre webpage,^115^ and Statistical Horizons,^116^ which provide valuable guidance on SMART. However, many are prohibitively expensive for individual researchers, and expanding their access by making them affordable may increase SMART’s utilisation.

Re-randomisation, a key feature of SMART design, enables tailoring treatment to individual characteristics and response. In this review, 77.5% of studies re-randomised non-responders in the second phase, aligning with SMART’s deviation from a ‘one-size-fits-all’ approach.^4^ A few trials used a run-in phase before stage 1, potentially reducing resource use and treatment burden by restricting further interventions to non-responders. However, this may limit generalisability and introduce selection bias, as only those with a severe form of the disease are likely to be included.

### Reporting in SMART

The CONSORT^112^ and SPIRIT^111^ statements stipulate reporting guidelines for any trials and trial protocols, respectively. However, reporting was inadequate in identifying trials as SMART, allocation concealment, sample size estimation, analysis software, data missingness, and pilot studies informing the main trial. While registration details, blinding, and sample size estimation were adequately reported, over half incorrectly registered SMART as non-sequential assignment models, suggesting limited knowledge of the design. These reporting gaps are consistent with a previous finding.^7^

Although the CONSORT and SPIRIT reporting guidelines apply to most trials, SMART has features that these guidelines may not capture. We found an unclear reporting of key SMART features such as decision rules, tailoring variables, and critical decision points. Failing to objectively report these features hinders replication by other researchers and undermines the transparency of SMART. While some authors may argue that there are no clear reporting guidelines for reporting SMART features. Hampton and colleagues recently recommended guidelines for reporting SMART features, which include reporting demographic characteristics, attrition, missing data for every randomisation stage, tailoring variables, and effect size for each embedded adaptive intervention.^117^ These recommendations may be relevant and a good starting point. However, developing reporting guidelines should be systematic, thorough and involve an expert consensus meeting and/or Delphi process.^118,119^

### External data in SMART

Given the novelty of SMART in health research, it is not surprising that only 21% of SMART used external data. While the cost and time-saving potentials are well documented in standard trials,^120,121^ no prior review, to the best of our knowledge, has examined its use in SMART. Standard RCTs commonly use external data from registries, EHRs, cohorts and administrative databases,^14,15,122–125^ supported by reporting guidelines.^23^ One explanation is SMART’s methodological complexity, which complicates integrating observation data, especially when detailed treatment adaptations are needed to inform decision rules.^8^ Investigators may also lack experience and guidance on leveraging external data within SMART. The absence of methodological and reporting guidelines further hinders this integration.

Beyond design complexity, barriers common to standard RCTs may also apply to SMART. These include limited access to external data due to bureaucracy, data security, and privacy concerns^126^ and infrastructure gaps such as weak data governance frameworks and IT support.^121^ In addition, statistical challenges – such as handling missing data, selection bias, and immortal time bias – require sophisticated methods,^127^ not always available to researchers.

Nevertheless, the minimal use of observation data in SMART is a missed opportunity. External data have only supplemented participant enrolment, outcome, and baseline covariate measurements. Because SMART design mirrors routine clinical practice, particularly in most chronic and recurrent diseases, rich longitudinal datasets from EHRs or registries hold potential for feasibility and efficiency.^5^ Such data could inform estimates of response rates, decision points, tailoring variables, and improve sample representativeness.^128^ External data can also help identify adaptive interventions. For example, Krakow et al.^129^ used a registry and Q-learning,^130^ to derive adaptive interventions that may then be prospectively validated in a SMART.

### Strengths and limitations

This review provides the most comprehensive synthesis of completed SMART and protocols. Our search identified 80 studies, double the number found in the previous review. We systematically evaluated trial characteristics, reporting practices and use of external data, offering new insights into methodological gaps and under-utilisation of real-world data in SMART. This is the first review to examine external data in SMARTs, highlighting an important design consideration. Furthermore, we offer a detailed record of SMART features such as re-randomisation, decision rules and tailoring variables, contributing to the need for tailored reporting guidelines.

This review has limitations. We excluded non-English publications and unpublished protocols, which may have introduced selection and publication bias. The focus on published trials may also overrepresent trials with favourable results or clearer reporting practices. While we identified whether external data were used, we did not assess their quality, completeness, or analytic contribution of these sources to trial design or outcomes, which remains an important area for future investigation. In addition, the interpretation of SMART features was limited by the inconsistent or incomplete reporting, underscoring the need for standardised methodological guidance and reporting standards.

## Conclusion

The SMART design is an innovative and increasingly used methodology in clinical research, with application across diverse conditions. However, key methodological features unique to SMART remain inconsistently reported and underreported, limiting transparency, reproducibility, and translation. Although external data such as EHRs, disease registries, and cohort studies are common in traditional RCTs, their use in SMART remains limited. This gap likely reflects methodological challenges, including data access and safety concerns, infrastructural hurdles, and a lack of reporting guidelines. Addressing these gaps will improve efficiency, enhance generalisability, reduce duplications and support translation of adaptive interventions.

To advance methodological quality, expert-led and consensus-driven reporting standards are needed, including extensions of CONSORT and SPIRIT. In parallel, guidance for reporting external data in SMART designs is required. Future research should examine which external data best support SMART, not only to inform tailoring variables, decision rules, and decision points, but also to identify candidate adaptive interventions. Equally important is the need for continuous affordable training programmes on SMART methodologies and interdisciplinary collaboration, particularly between biostatisticians and data scientists. Finally, given the complexity of SMART designs, journals could offer extra space or structured templates to support comprehensive reporting.

## Supplemental Material

sj-docx-1-ctj-10.1177_17407745251385535 – Supplemental material for A review of use of external data and update on reporting standards in Sequential Multiple-Assignment Randomised Trials Supplemental material, sj-docx-1-ctj-10.1177_17407745251385535 for A review of use of external data and update on reporting standards in Sequential Multiple-Assignment Randomised Trials by Isaac J Egesa, Laura Bonnett, Richard Emsley, Anthony Marson and Catrin T Smith in Clinical Trials
