# Supplementary material for: A review of use of external data and update on reporting standards in Sequential Multiple-Assignment Randomised Trials
Source: Clin Trials. 2025 Nov 5;23(1):85–98. doi: 10.1177/17407745251385535 (PMC12909610; doi:10.1177/17407745251385535)
Supplement: sj-docx-1-ctj-10.1177_17407745251385535 – Supplemental material for A review of use of external data and update on reporting standards in Sequential Multiple-Assignment Randomised Trials [file sj-docx-1-ctj-10.1177_17407745251385535.docx]

# APPENDIX 1: SEARCH STRATEGY

| Database |  | Search terms |
| --- | --- | --- |
| OVID MEDLINE | 1 | “adaptive intervention*” |
|  | 2 | “adaptive treatment strateg*” |
|  | 3 | “sequential multiple assignment randomi*” |
|  | 4 | “multistage treatment strateg*” |
|  | 5 | “dynamic treatment strateg*” |
|  | 6 | “stepped care intervention*” |
|  | 7 | “stepped care treatment*” |
|  | 8 | “just-in-time adaptive intervention" |
|  | 9 | “dynamic treatment regim*" |
|  | 10 | “multiple treatment cours*” |
|  | 11 | “proportionate intervention*” |
|  | 12 | “multi-course clinical trial” |
|  | 13 | “smart design*” |
|  | 14 | #1 OR #2 OR #3 OR #4 OR #5 OR #6 OR #7 OR #8 OR #9 OR #10 OR #11 OR #12 OR #13 |
|  | 15 | #14 AND limit 2 to (english language and yr="2020 -Current") |
|  |  |  |
| SCOPUS |  | ( TITLE-ABS-KEY ( "adaptive intervention*" ) OR TITLE-ABS-KEY ( "adaptive treatment strateg*" ) OR TITLE-ABS-KEY ( "smart design*" ) OR TITLE-ABS-KEY ( "multistage treatment strateg*" ) OR TITLE-ABS-KEY ( "dynamic treatment strateg*" ) OR TITLE-ABS-KEY ( "stepped care intervention" ) OR TITLE-ABS-KEY ( "stepped care treatment" ) OR TITLEABS-KEY ( "just-in-time adaptive intervention" ) OR TITLE-ABS-KEY ( "dynamic treatment regim*" ) OR TITLE-ABS-KEY ( "multiple treatment cours*" ) OR TITLE-ABS-KEY ( "proportionate intervention*" ) OR TITLEABS-KEY ( "sequential multiple assignment randomi*" ) ) AND ( EXCLUDE ( SUBJAREA , "BUSI" ) OR EXCLUDE ( SUBJAREA , "CENG" ) OR EXCLUDE ( SUBJAREA , "ENGI" ) AND PUBYEAR > 2020 |
|  |  |  |
| WEB OF SCIENCE |  | TS=(“proportionate intervention*”) OR TS=(“multiple treatment cours*”) or TS=(“dynamic treatment regim*") or TS=(“just-in-time adaptive intervention") or TS=(“stepped care treatment") or TS=(“stepped care intervention*”) or TS=(“dynamic treatment strateg*”) or TS=(“multistage treatment strateg*”) or TS=(“sequential multiple assignment randomi*”) or TS=(“adaptive treatment strateg*”) or TS=(“adaptive intervention*”) and Preprint Citation Index (Exclude – Database) and 2024 or 2023 or 2022 or 2021 or 2020 (Publication Years) and English (Languages) |
|  |  |  |
| PsycINFO |  | “adaptive intervention*” OR “adaptive treatment strateg*” OR “sequential multiple assignment randomi*” OR “multistage treatment strateg*” OR “dynamic treatment strateg*” OR “stepped care intervention*” OR “stepped care treatment*” OR “just-in-time adaptive intervention*" OR “dynamic treatment regim*" OR “multiple treatment cours*” OR “proportionate intervention*” OR “smart design*”. Filter by english langauge and year of publication 2020 - current |
|  |  |  |
| PUBMED |  | ("adaptive intervention*"[All Fields] OR "adaptive treatment strateg*"[All Fields] OR "sequential multiple assignment randomi*"[All Fields] OR "multistage treatment strateg*"[All Fields] OR "dynamic treatment strateg*"[All Fields] OR "stepped care intervention"[All Fields] OR "stepped care treatment"[All Fields] OR "just-in-time adaptive intervention"[All Fields] OR "dynamic treatment regim*"[All Fields] OR "multiple treatment cours*"[All Fields] OR "proportionate intervention"[All Fields] OR "SMART design"[All Fields]) AND ((english[Filter]) AND (2020:2024[pdat])) |

# APPENDIX 2A: COMPLETED SMART TRIALS

| Author | country | Medical condition | Randomisation phases | Interventions per stage | Tailoring variable | Primary outcomes | Use of external data |
| --- | --- | --- | --- | --- | --- | --- | --- |
| Bianchi S^1^ | Italy | Cancer | Two | **Phase I:** docetaxel 4 cycles with maintenance Vs. suspension of androgen deprivation therapy (ADT) **Phase II**: continue with docetaxel to complete 10 cycles Vs. interrupt docetaxel until PSA rose by 50% (10ngmL) for responders and continue with phase I intervention for non-responders. | > 50% decrease in prostate-specific antigen (PSA) from baseline in two or more PSA measurements obtained at least 4 weeks apart | Survival | No |
| Cinciripini P^2^ | USA | smoking cessation | Two | **Phase I:** standard dosage varenicline Vs. combined nicotine replacement therapy **Phase II**: continue with phase I intervention for responders and switching Vs. continuing Vs. dosage increase for non-responders | 7-day point prevalence with expired carbon monoxide (CO) <6 ppm) | Point prevalence of a seven-day abstinence | No |
| Fatori D^3^ | Brazil | Obsessive-compulsive disorder | Two | **Phase I**: fluoxetine Vs. group cognitive behaviour therapy **Phase II**: continue with phase I intervention for responders and switch Vs. augment for non-responders | 50% reduction in baseline YBOCS (Yale-Brown Obsessive-Compulsive Scale) scores and CGI (Clinical Global Impression) scores of 1 or 2 | Obsessive-compulsive score using YBOCS | No |
| Fortney J^4^ | USA | Post-traumatic disorder & bipolar | Two | **Phas**e I: telepsychiatry collaborative care Vs. telepsychiatry enhanced referral **Phase II**: continue with intervention for telepsychiatry enhanced referral arm for responders while telepsychiatry collaborative care is not assessed for response. telepsychiatry enhanced referral Vs. Phone-Psychiatry Referral for non-responders. | ≤2 interaction videos in the first 6 months | Mental Health-Related Quality of Life using Short Form 12 Mental Health Composite Summary (MCS) | No |
| Friedman B W^5^ | USA | Musculoskeletal pain | Two | **Phase I**: acetaminophen Vs. no initial treatment **Phase II**: Ibuprofen Vs. ketorolac 20mg for non-responders | Responding “no” when asked if they needed additional pain medication | Pain score using 11-point numerical scale defined as a 1.3-unit improvement on a scale 0-10 | No |
| Fu S S^6^ | USA | Smoking | Two | **Phase I**: four Vs. eight-week tobacco longitudinal care **Phase II**: monthly tobacco longitudinal care Vs. quarterly tobacco longitudinal care for responders and tobacco longitudinal care Vs. tobacco longitudinal care + medication therapy management for non-responders | No smoking in the last 7 days | Prevalence of self-reported long-term abstinence | No |
| Gao K^7^ | USA | Bipolar | Two | **Phase I**: Lithium Vs. divalproex **Phase II**: Switch vs. switch + quetiapine Vs. switch + lamotrigine | A CGI score of ≤ 2 plus tolerability on the assigned medication | Time to early termination and time to termination after randomisation | No |
| Geng E H^8^ | Kenya | HIV | Two | **Phase I:** standard of care (SOC) Vs. short message service (SMS) Vs. conditional cash transfer (CCT) **Phase II:** continue Vs. stop for responders and SOC + outreach Vs. SMS + CCT Vs. peer navigation | Retention in care defined as missed clinic visit < 14 days from appointment date in a 1-year follow-up | Proportion retained in care | No |
| Grilo C M^9^ | USA | Eating disorder (binge eating) | Two | **Phase I:** six months of behavioural weight loss (BWL) Vs. one month of stepped care BWL **Phase II**: BWL + medication Vs. BWL + placebo for responders and cognitive behaviour therapy (CBT) + medication Vs. CBT + placebo for non-responders | ≥65% decrease in binge eating at 1 month from baseline assessment | Binge eating remission and proportion of weight loss | No |
| Hammel P^10^ | France | Cancer | Two | **Phase I**: gemcitabine Vs. Gemcitabine + erlotinib **Phase II**: Chemotherapy vs. chemoradiotherapy | Controlled tumour or stable with objective response and had WHO (World Health Organisation) performance status of ≤2 after 4 months | Survival | No |
| Karp J.F^11^ | USA | Knee osteoarthritis with comorbid depressive symptoms | Two | **Phase I:** enhanced usual care (EUC) + cognitive behaviour therapy (CBT) Vs EUC Vs. EUC + Physical therapy (PT) **Phase II:** CBT Vs. PT | Much better or very much better on the P-GIC (Patient Global Impression of Change) scale assessed quarterly | Pain score using P-GIC | yes |
| Kasari C^12^ | USA | Autism | Two | **Phase I**: Joint Attention Symbolic Play (JASP) + Enhanced Milieu Teaching (EMT) Vs. JASP + EMT + Speech Generating Device (SGD) **Phase II**: Increased sessions Vs. added SDG | ≥25% change in at least half of the variables at 12 weeks | Number of communicable utterances | No |
| Kim JS^13^ | USA | Learning disorder | Two | **Phase I**: Conceptual coherent texts (CCT) + App Vs. Levelled Texts (LT) + App **Phase II**: Augment only Vs. Augment + Intensify for non-responders | Completion of all App activities for at least one book measured in two to four weeks | Academic progress using a measure of academic progress (MAP) | No |
| Lebeau B^14^ | France | Cancer | Two | **Phase I**: aspirin + chemotherapy Vs. chemotherapy only **Phase II**: continue with chemotherapy vs no further intervention for responders | Response to an intervention by the end of 6^th^ course of chemotherapy using WHO criteria | Survival | No |
| Matthay K^15^ | USA | Cancer | Two | **Phase I**: consolidation with myeloablative chemotherapy, total-body irradiation, and autologous purged bone marrow transplantation (ABMT) VS. 3-cycles of intensive chemotherapy **Phase II**: no further treatment Vs. 13-cis-retinoic acid | Complete response or partial  Response based on the International Neuroblastoma Response criteria | Event free survival | No |
| McKay J. R^16^ | USA | Alcohol & cocaine dependence | Two | **Phase I**: run-in intensive outpatient program (IOP) then motivation interviewing intensive outpatient program (MI-IOP) Vs. motivation interviewing patient choice (MI-PC) **Phase II**: MI-PC Vs. no further outreach care (NFC) | Engagement (< 2 sessions missed) in IOP in the first two to eight weeks | Alcohol and drug use 30-day point prevalence | No |
| Morgenstern J^17^ | USA | Alcohol use disorder | Two | **Phase I**: run-in brief advice (BA) then BA + one session Vs. motivational interviewing (MI) **Phase II**: MI Vs. MI + behavioural self-control training (BSCT) | Reduced drinking to NIAAA (National Institute on Alcohol Abuse and Alcoholism) safe drinking guidelines after 4 weeks | Total standard drinks per week and total number of heavy drinking days | No |
| Morin C M^18^ | Canada | Insomnia | Two | **Phase I**: zolpidem Vs. behaviour therapy (BT) **Phase II**: zolpidem Vs. cognitive therapy (CT) and trazodone Vs. BT | ≥8-point reduction on ISI (Insomnia Severity Index) score at 6 weeks compared to baseline. | Proportion of treatment response and remission rates defined using insomnia severity index total score | No |
| Naar-King S^19^ | USA | Obesity | Two | **Phase I**: home-based motivational interviewing (HB-MIS) Vs. office-based motivational interviewing (OB-MIS) **Phase II**: motivational interviewing (CM) Vs. standard of care | Weight loss of ≥3% at 3 months post-baseline randomisation weight | Proportion of overweight | yes |
| Patrick M E^20^ | USA | Alcohol use disorder | Two | **Phase I**: assessment-only Control Vs. early stage 1 intervention Vs. late stage 1 intervention **Phase II**: resource email Vs online health coach | Having ≥2 episodes of ≥4/5 drinks for female/male at 2 weeks post-randomisation or ≥1 episode of ≥8/9 drinks for female/male at 2 weeks post-randomisation | Total number of binge drinking | No |
| Pelham W. E^21^ | USA | Attention deficit hyperactivity disorder | Two | **Phase I**: low dose medication Vs. low dose cognitive behaviour therapy **Phase II**: augment Vs. combine interventions | < 75% ITBE (Individualised target behaviour evaluation) performance assessed using IRS (Impairment Rating Scale) at 8 weeks post-randomisation | Total number of observed classroom violations | No |
| Petracci E^22^ | Italy | Cancer | Two | **Phase I**: chemotherapy (CT) only then bevacizumab Vs. CT + bevacizumab then CT only **Phase II**: CT Vs. CT + Cetuximab and CT + bevacizumab Vs. CT + bevacizumab + Cetuximab | Presence of Kras gene | Survival | No |
| Petry N.M^23^ | USA | Cocaine use disorder | Two | **Phase I**: usual standard care (UC) Vs. contingency management (CM) **Phase II**: UC Vs. CM Vs. no intervention | None | Total number of days attended treatment, proportion of scheduled group attended, duration of consecutive treatment attendances and proportion of negative drug sample submitted. | No |
| Powell B. L^24^ | USA | Acute promyelocytic leukemia | Two | **Phase I**: Standard induction & consolidation therapy with 25-day courses of Arsenic oxide Vs. Standard induction & consolidation therapy without 25-day courses of Arsenic oxide **Phase II**: Responders assigned to ATRA maintenance + oral 6-mercaptopurine + oral methotrexate Vs. ATRA maintenance alone | Complete remission after stage one randomisation or consolidation therapy | Event free survival | No |
| Sinyor M^25^ | USA | Depression | Four | **Phase 1**: Run-in citalopram (CIT) then compare four switch options (sertraline(SER), bupropion(BUP), venlafaxine(VEN) and cognitive therapy(CT)) Vs. three augmentation options (CIT + BUP), buspirone(BUS + CIT), and CIT + CT). **Phase II**: Switch options VEN Vs. BUP for non-responders in CT and CIT + CT **Phase III**: Switch options (Mirtazapine (MRT), nortriptyline(NTP)) Vs. augment options (Lithium(Li) or thyroid hormone(THY)) for non-responders in phase I **Phase IV**: switch options (tranylcypromine(TCP) Vs. augment (MRT-VEN combination) for non-responders | ≥50% reduction in QIDS-SR (Quick Inventory of Depressive Symptomatology-Self Report) score post-previous randomisation stage | Remission rate defined using clinically rated 17-item Hamilton scale of depression | No |
| Schlam T R^26^ | USA | Smoking cessation | Two | **Phase I**: Run-in quit treatment then non-responders randomised to preparation treatment Vs. recycling treatment Vs. quitline referral **Phase II**: Factorial supportive counselling Vs. skills training | Smoking for ≥ 7consecutive days at 6 months post-baseline randomisation | Point prevalence of a seven-day abstinence | yes |
| Schmitz J M^27^ | USA | Cocaine use disorder | Two | **Phase I**: Acceptance and commitment (ACT) + Contingency management (CM) Vs. Drug counselling (DC) + CM **Phase II**: Add placebo Vs. Add madafinil | ≥6 consecutive cocaine-negative urine within two weeks at week four post-stage one randomisation | Proportion of negative cocaine tests in a urine sample | No |
| Sherwood N E^28^ | USA | Weight loss (obesity) | Two | **Phase I**: Three Vs. seven weeks of Standard Behavioral Weight Loss (SBT) **Phase II**: Augment + portion-controlled meals Vs. Switch (Acceptance-Based Behavioural weight loss treatment) | ≥2.5% and ≥5% weight loss post-stage 1 randomisation after three and seven weeks, respectively | Mean weight loss | No |
| Sikorskii A^29^ | USA | Cancer | Two | **Phase I**: Symptom management and survivorship handbook (SMSH) Vs. Telephone Interpersonal Counselling (TIP-C) + SMSH **Phase II**: SMSH Vs SMSH+TIP-C for non-responders in SMSH arm | Improvement in weekly GSDS (General Symptom Distress Scale) score from worse stage to better stage at week 4 post stage one randomisation | Symptom Severity Index defined using GSDS | No |
| Smith S N^30^ | USA | Depression & Anxiety | Two | **Phase I**: Replicating Effective Programs (REP) Vs. REP + coaching **Phase II**: REP + coaching Vs. REP + coaching + facilitation Vs. REP + facilitation | At least one school professional delivering <3 CBT (Cognitive behaviour therapy) to < 10 students or school professionals reporting >2 barriers to CBT | Total number of cognitive behaviour therapy sessions delivered | No |
| Somers T J^31^ | USA | Pain coping skills | Two | **Phase I**: Five Vs. one session of Pain coping skills training (PCST) **Phase II**: Continue Vs. stop Vs. Increase dosage | ≥30% pain reduction to the baseline pain score between weeks five and eight after stage 1 randomisation |  | Yes |
| Swartz M. S^32^ | USA | Schizophrenia | Three | **Phase I**: First (perphenazine) Vs. second-generation antipsychotic medications (Olanzapine vs quetiapine vs risperidone vs ziprasidone) **Phase II**: Olanzapine Vs. quetipine Vs. Risperidone for first-generation failures **Phase III**: Olanzapine Vs. risperidone Vs. quetipine Vs. clozapine failure due to efficacy and Olanzapine Vs. risperidone Vs. quetipine Vs. ziprasidone for failure due to side effects | Inefficacy, side effects and patients’ dissatisfaction to randomised drug | Treatment discontinuation rate | No |
| Thall P. F^33^ | USA | Prostate cancer | Two | **Phase I**: cyclophosphamide, vincristine, and dexamethasone (CVD) Vs. ketoconazole plus doxorubicin alternating with vinblastine plus estramustine (KA/VE) Vs. paclitaxel, estramustine, and carboplatin (TEC) Vs. paclitaxel, estramustine, and etoposide (TEE) **Phase II**: Switch to non-randomised intervention | ≥40% PSA reduction at 8 weeks post-baseline PSA levels | Proportion of responders | No |
| Tummarello D^34^ | Italy | Cancer | Two | **Phase I**: cyclophosphamide doxorubicin vincristine with Teniposide (CAV-T) vs cyclophosphamide doxorubicin vincristine with Etoposide (CAV-E) **Phase II**: rIFN-alpha-2b, 3 M.U Vs. no therapy | A complete response to first-stage randomised intervention | Survival | No |
| Wyatt G^35^ | USA | Cancer | Two | **Phase I**: Reflexology Vs. meditative Vs. placebo **Phase II**: Continue Vs. combined | No improvement in fatigue symptoms on the MDASI (M.D. Anderson Symptom Inventory) scale at 4 weeks post stage one randomisation | Fatigue severity score using Brief Fatigue Inventory and Attention Functional Index | No |

# APPENDIX 2B: SMART TRIAL PROTOCOLS

| **Author** | **Country** | **Medical condition** | **Randomisation phases** | **Interventions per stage** | **Response definition** | **Primary outcomes** | **Use of external data** |
| --- | --- | --- | --- | --- | --- | --- | --- |
| Abuogi L.^36^ | Kenya | HIV | Two | Phase I: Standard of care Routine education and counselling (SOC-REC) Vs. Electronic Navigation (e-NAV) Phase II: SOC-Outreach and Intensified Counselling (SOC-OIC) Vs. Conditional Cash Transfer (CCT) Vs. In-person Navigation | Viral suppression >1000 copies and missed clinic visits >14 days | Proportion with viral suppression & adherence | Yes |
| Arean P^37^ | USA | Depression | Two | **Phase I**: message-based psychotherapy (MBP) Vs. videoconferencing-based psychotherapy (VBP) **Phase II**: weekly MBP + VBP Vs. monthly MBP + VBP | <50% reduction in PHQ-9 (Patient Health Question) score from baseline | PHQ-9 and Social Life and Family Life Scale of Sheehan Disability Scale (SDS)score | No |
| Auyeung S F^38^ | USA | Depression | Two | Phase I: Escitalopram Vs. Methylphenidate (MPH) Phase II: Switch Vs. combine phase I interventions | ≥11 score on Hamilton Depression Rating scale | Proportion adhered to IFN-alpha treatment | Yes |
| Bahraini N H^39^ | USA | Suicide | Two | Phase I: Implementation as usual care (IAU) vs Audit & Feedback Phase II: Continue phase I intervention Vs. augment with facilitation | ≥80% on C-SSRS (Columbia-Suicide Severity Rating Scale) & CSRE (comprehensive suicide risk evaluation) scale or <20% dropouts | Proportion of C-SSR and CSRE uptake | Yes |
| Becker K^40^ | Germany | ADHD | Two | **Phase I**: Low Telephone Assisted Self Help (TASH) Vs. waitlist control **Phase II:** Parent Management and Preschool Teacher Training (PMPTT) Vs. treatment as usual | Clinician rate score of ≥0.7 based on ODD | Clinician-rated ADHD and ODD score | No |
| Belzer M^41^ | USA | HIV | Two | **Phase I**: cell phone support (CPS) vs short message service (SMS) **Phase II**: CPS + incentives Vs SMS + incentives | <200 copies/ml | Viral suppression | Yes |
| Buchholz S.^42^ | USA | Physical activity | Two | Phase I: Enhanced physical activity monitoring Vs. enhanced physical activity monitoring + text messaging  Phase II: Personal calls Vs. group meetings | Fail to wear Fitbit at least 3 out of 7 days in weeks 6 to 8, with either 10 hours of wear time per day or exceeding their baseline step average for that day or average steps not exceeding short-term goal of 600 steps above the baseline average for a day | Average daily step count | No |
| Carr E^43^ | Ireland | Stroke | Two | Phase I: Structured exercise Vs. lifestyle physical activity Phase II: Combine Vs. Switch phase I intervention | ≥5% change in average weekly step count | Average daily step count | No |
| Comins C^44^ | South Africa | HIV | Two | Phase I: Decentralised treatment program (DTP) Vs. Individualised case management (ICM) Phase II: Switch Vs. combine phase I interventions | Viral suppression <50 copies/Ml | Composite outcome of retention in ART care & proportion of HIV viral suppression | Yes |
| Drake C^45^ | USA | Insomnia, Major depression disorder | Two | Phase I: Digital CBT-Insomnia Vs. online sleep education Phase II: Continue Phase I Intervention Vs. clinician-led CBT-I Vs. sleep education | >7 score on the Insomnia severity index | Insomnia score on the ISI (Insomnia Severity Index) scale | Yes |
| Edelman E J^46^ | USA | Tobacco use disorder | Two | Phase I: Nicotine replacement therapy (NRT) with contingency management (CM) Vs. NRT alone Phase II: Switch to varenicline + CM Vs. Augment | Self-reported abstinence and eCO ≤6ppm | Proportion of self-reported smoking abstinence confirmed by eCO | Yes |
| Eldridge E D^47^ | USA | Insomnia | Two | **Phase I:** online CBT (cognitive behaviour therapy) - insomnia + usual positive airway pressure (PAP) care Vs. Usual PAP **Phase II:** Therapist-led CBT Vs. continued online CBT- insomnia and PAP + Hygiene Vs PAP | <8 scores on the insomnia severity index | Insomnia remission rates | No |
| Fernadez M^48^ | USA | Tobacco use disorder | Three | Phase I: Ask for advice on Connect (AAC)-in Vs. AAC-out Phase II: Text messaging (TM) Vs. continued AAC  Phase III: Continue TM Vs. TM + MAPS (motivation and problem-solving) | Enrolment in Utah Tobacco Quit Line (UTQL) | Composite outcome: Reach defined as the proportion of tobacco users who enter quitline treatment; Impact defined as Reach*efficacy [efficacy defined as the proportion of tobacco users entering quitline treatment who quit]; the proportion of abstinence from tobacco. | No |
| Flynn D^49^ | USA | Chronic pain | Two | **Phase I:** Standard Rehabilitation Care (SRC) Vs. Complimentary and Integrative Health (CIH) **Phase II:** Switch vs. combined intervention | ≥ 3-point score on pain impact score after 3 weeks | Pain impact score on PROMIS scale | No |
| Fox C K^50^ | USA | obesity | Two | Phase I: 12 Vs. 24 weeks lifestyle therapy (LST) Phase II: Switch duration + LST + phentermine + topiramate Vs. switch duration + LST + topiramate | ≥5% BMI reduction | Percentage change in BMI | Yes |
| Fritz J M^51^ | USA | Low back pain (chronic pain) | Two | Phase I: Physical therapy (PT) vs Army Medicines’ holistic Move2Health (M2H) Phase II: Combined phase I intervention Vs. Mindfulness Oriented Recovery Enhancement (MORE) program | ≥7 T score on PI-CAT (pain interference computer-adapted test) scale from baseline score | Pain score on PROMIS (patient-reported outcomes measurement information system) and PI-CAT (pain interference computer-adapted) scales | No |
| Germeroth L J^52^ | USA | obesity | Two | Phase I: Health & Behavioural Transition (HABITpreg) Vs. Treatment as usual (TAUpreg) Phase II: HABITpost Vs. TAUpost | None | Maternal weight | No |
| Hasset A L^53^ | USA | Chronic low back (Chronic pain) | Two | **Phase I:** Run-in web-based pain self-assessment programme followed by physical therapy or exercise Vs. mindfulness-based stress reduction (MBSR) Vs. acupressure Vs. duloxetine **Phase II:** switch phase I interventions | ≥2 scores change on patient global impression after 8 weeks post-baseline | Pain score on PROMIS scale | No |
| Heerden A V^54^ | South Africa | HIV | Two | Phase I: Standard of care (SOC) Vs. SOC + Lottery incentives Phase II: Continue Vs. smart locker ART pick-up vs home delivery | Detectable viral load or no retention at six months | Proportion with viral suppression | No |
| Hibbard J C^55^ | USA | Hypertrophic burn scar (Plastic surgery) | Three | Phase I: Pulsed-dye laser (PDL) Vs. CO2 laser Vs. MED Phase II: Switch Vs, continue phase I intervention | none | Vancouver Scar score | No |
| Johnson J^56^ | USA | Depression | Two | **Phase I:** Run-in enhanced implementation as usual (EIAU) followed by low-intensity coaching and feedback (LICF) + EIAU Vs. EIAU only **Phase II:** Continue Vs. augment with High-intensity coaching and feedback (HICF) | No planned intervention and or clinical failure to sustain at subsequent assessments | Proportion sustained core ROSE (Reach Out, Stay Strong, Essentials for mothers of newborns) elements | No |
| Kilbourne A^57^ | USA | Depression | Two | Phase I: Run-in replication effective program (REP) followed by REP + EF (External facilitation) Vs. REP + EF + IF (Internal facilitation) Phase II: Continue Vs. Augment with IF for REP + EF arm | <50% of participants receiving <3 life goal sessions | Health-related quality of life score and mood disorder score on PHQ-9 questionnaires | No |
| Kopelowicz A^58^ | USA | Diabetes | Two | Phase I: Community worker-led group session Vs. Nurse-led sessions Phase II; Multifamily Group Vs Stepped up Phase I intervention | ≥50% gain on summary of diabetes self-care activities (SDSCA) in 3rd session after 6 weeks | Diabetes self-care activities score using the SDSCA (Summary of Diabetes Self-Care Activities) scale | No |
| Kor P P K^59^ | Hongkong | Dementia | Two | **Phase I**: Behavioural activation (BA) Vs. mindfulness practice (MP) **Phase II**: continue phase I intervention Vs. Augment with self-efficacy enhancing strategy | ≥50 reduction in PHQ-9 score from baseline score | Depressive symptoms score on PHQ-9 | No |
| Levy R^60^ | Kenya | Major depressive disorder & PTSD | Two | Phase I: Interpersonal psychotherapy (IPT) Vs. fluoxetine Phase II: Switch Vs. combine phase I intervention | No diagnosis of major depressive disorder (MDD) using M.I.N.I scale at three and six months | Proportion of MDD and post-traumatic stress disorder (PTSD) diagnosis and depression scores using Beck Depression Index (BDI) and post-traumatic stress checklist scales | No |
| Li X^61^ | China | Schizophrenia | Two | **Phase I:** Olanzapine Vs. Risperidone Vs. Amisulpride Vs. Aripiprazole Vs. Perphenazine **Phase II:** Switch to Olanzapine Vs. Amisulpride Vs. Clozapine **Phase III:** Augment with MECT Vs. clozapine extended treatment Vs. switch to clozapine in the non-clozapine arm and other second-generation antipsychotics (SGAs) | ≥40% score reduction on Positive and Negative Syndrome Scale (PANSS) after eight weeks | PANSS score | No |
| Liu H^62^ | China | Suicide | Two | **Phase I:** Monthly Brief Contact Intervention (BCI) Vs. weekly BCI **Phase II**: Step down BCI Vs continue for responders and step up BCI Vs. continue for non-responders | Increased suicide risk from baseline risk three months post-randomisation measured using M.I.N.I-suicidality | Suicide ideation score | No |
| Mustanski B^63^ | USA | HIV | Two | **Phase I:** Run-in SMART sex education (SSE) followed by SSE + booster I Vs. SMART Squad + Booster I **Phase II**: Continue Vs. Augment SMART squad booster II | 100% condom use and condom intentions score ≥3.76 and self-efficacy score ≥6.50 at six months | Total number of condomless anal sex partners from most three recent partners, condom use intention score and HIV testing | No |
| Nelson B^64^ | Australia | Psychotic disorders | Three | **Phase I:** Run-in support & problem-solving skills (SPS) sessions followed by monitoring Vs. maintenance or relapse prevention SPS **Phase II:** Cognitive Behavioural Case Management (CBCM) Vs. SPS **Phase III**: CBCM + Selective Serotonin Reuptake Inhibitor (SSRI) Vs. CBCM + Placebo | score of <3 for all four positive symptoms at weeks four and six for Phase I and weeks 12 and 24 for Phase II | Global functioning scale score 6 months from baseline and end of step 2 | No |
| O'keefe V M^65^ | USA | Suicide | Two | Phase I: New hope Vs. optimised case management Phase II: Elders resilience + optimised case management Vs. optimised case management | none | Suicide ideation score on the SIQ-JR (Suicide Ideation Questionnaire Junior) scale and resilience score on the RSEA (Resiliency Scales for Children and Adolescents) scale | No |
| Osilla K C^66^ | USA | Alcohol use disorder | Two | Phase I: Partners connect Vs. Gottman resources Phase II: CRAFT workbook Vs. phone CRAFT Vs. partners connect | Completion of personalised normative feedback after two months | Total number of drinks per week in the past one-month and proportion seeking health through SMART intervention | No |
| Peter S C^67^ | USA | Opioid use disorder | Two | **Phase I**: Contingency measures (CM) Vs. BSM (brief motivational interviewing plus substance-free activities session plus mindfulness) **Phase II:** Combine Vs. Switch phase I intervention | Attending physician appointments and positive buprenorphine in the e-sample | Medication adherence proportion | No |
| Peterson B S^68^ | USA | Anxiety disorder | Two | **Phase I**: Fluoxetine Vs. cognitive behaviour therapy (CBT) **Phase II:** Optimise Phase I Intervention Vs. combine | Screen for Child Anxiety Related Disorder (SCARED)-41 score below the diagnostic threshold for any single anxiety disorder and total youth SCARED-41 score <10 and <8 score for child anxiety impact scale | Youth SCARED (Screen for child anxiety-related disorders) score | No |
| Quanbeck A^69^ | USA | Opioid use disorder | Two | **Phase I**: Run-in educational/engagement meeting (EM) and Audit with monthly feedback (AF) followed by EM & AF + practice facilitation (PF) Vs. EM & AF only **Phase II**: Augment Phase I with prescriber peer consulting (PPC) | none | Morphine-milligram equivalent (MME) dose by prescribing clinicians within clinics | Yes |
| Rabin B A^70^ | USA | COVID-19 | Two | Phase I: mHealth outreach Vs standard of care (SOC) Phase II: Augment with care coordination Vs. Continue phase I intervention | Unable to update COVID-19 vaccination status or have at least one outstanding preventive service need | Proportion of complete COVID vaccination | Yes |
| Sabri B^71^ | USA | Intimate partner violence | Two | **Phase I:** Online safety decision Vs. usual care **Phase II:** Text message Vs. text message + phone call | No improvement in safety (significant reduction in severity & frequency of IPV) and empowerment scores <0.25 SD of the average score | Safety score using conflict tactics scale and empowerment score using personal progress scale | No |
| Smith S K^72^ | USA | PTSD | Two | Phase I: Cancer distress coach (CaDC) app Vs. Usual care Phase II: CaDC app + coaching Vs. cognitive behaviour therapy | ≥5 PTSD checklist score improvement after four weeks | PTSD score on PTSD Checklist for DSM5 scale | Yes |
| Sripada R K^73^ | USA | PTSD | Two | Phase I: Clinician-supported (CS) coach Vs. PE-PC (prolonged exposure-primary care) Phase II: Continue phase intervention Vs. Step-up phase I intervention | Response defined based on PCL-5 >60-slow responder and <29-early responders | Clinician administered a PTSD severity score on CAPS-5 scale | No |
| Velloza J^74^ | South Africa | HIV | Two | **Phase I**: Standard of care (SOC) PrEP counselling + two-way SMS Vs. SOC PrEP counselling + WhatsApp group adherence support **Phase II:** Augment with quarterly drug-level feedback counselling Vs. monthly issue-focused counselling | Tenofovir diphosphate (TFV-DP) levels ≥500fmol/punch after 2 months | Proportion of PrEP adherence | No |
| Walton M A^75^ | USA | Alcohol use disorder & Violent behaviours | Two | **Phase I**: Text message Vs. health coach phoning intervention **Phase II**: Continue phase I Vs. resource brochure for responders and augment phase I with personalised messages Vs. switching for non-responders | Self-report of binge drinking or aggression in weeks three and four. | Total number of alcohol consumptions in the past 30 days | No |
| Wan Y^76^ | China | Dementia | Two | **Phase I**: monthly Online Vs monthly physical visits **Phase II**: step down to bi-monthly for responders Vs. Augment for non-responders to bi-weekly | ≥10 points on Quality of Life in Alzheimer's Diseases compared to the baseline score and reduction in caregivers' burden from the baseline using Zarit Burden Interview | Proportion of people demonstrating an improvement in QoL (QoL-Alzheimer disease) and proportion of caregivers exhibiting a reduction in caregiver burden (Zarit Burden Interview-ZBI) | Yes |
| Windsor L^77^ | USA | COVID-19 | Two | Phase I: Brochure vs navigation Phase II: Continue phase I intervention Vs. brief counselling Vs. critical dialogue | A COVID-19 test after 7 days | Proportion of completed COVID-19 tests | No |
| Zhao S Z^78^ | Hongkong | smoking cessation | Two | **Phase I**: Personalised instant messaging (PIM) Vs. regular instant messaging (RIM) **Phase II**: Combined Vs. Switch phase I intervention | Self-report of not smoking for ≥7 days after one month | Biochemical abstinence proportion | No |
| Zhou G^79^ | Kenya | Malaria | Two | Phase I: long-lasting insecticide-treated nets (LLINs) Vs. long-lasting piperonyl butoxide-treated nets (PBO LLIN) Vs. LLIN + Indoor residual spraying (IRS) Phase II: Augment with larval source management (LSM) Vs. continue phase I intervention | Change in the malaria incidence rate and the pre-defined threshold value of cost-effectiveness set by the Ministry of Health | Malaria incidence rate | No |
| Zullig L L^80^ | USA | Cancer | Two | **Phase I:** IGuide intervention to patients & PHC (primary health care) providers Vs. education-only control **Phase II**: continue phase I Vs. IGuide II with tailored videos and specialised consultant with PHC | ≥90% of participants not meeting modified HEDIS quality metrics at six months | HEDIS (Healthcare Effectiveness Data and Information Set) measures (Blood pressure, blood glucose), medication adherence proportion | Yes |

# Appendix 3: PRISMA checklist

| **Section and Topic** | **Item #** | **Checklist item** | **Location where item is reported** |
| --- | --- | --- | --- |
| **TITLE** | | |  |
| Title | 1 | Identify the report as a systematic review. | Abstract |
| **ABSTRACT** | | |  |
| Abstract | 2 | See the PRISMA 2020 for Abstracts checklist. | Abstract |
| **INTRODUCTION** | | |  |
| Rationale | 3 | Describe the rationale for the review in the context of existing knowledge. | 1-2 |
| Objectives | 4 | Provide an explicit statement of the objective(s) or question(s) the review addresses. | 1-2 |
| **METHODS** | | |  |
| Eligibility criteria | 5 | Specify the inclusion and exclusion criteria for the review and how studies were grouped for the syntheses. | 3-4 |
| Information sources | 6 | Specify all databases, registers, websites, organisations, reference lists and other sources searched or consulted to identify studies. Specify the date when each source was last searched or consulted. | 3-4 |
| Search strategy | 7 | Present the full search strategies for all databases, registers and websites, including any filters and limits used. | Supplementary |
| Selection process | 8 | Specify the methods used to decide whether a study met the inclusion criteria of the review, including how many reviewers screened each record and each report retrieved, whether they worked independently, and if applicable, details of automation tools used in the process. | 3-4 |
| Data collection process | 9 | Specify the methods used to collect data from reports, including how many reviewers collected data from each report, whether they worked independently, any processes for obtaining or confirming data from study investigators, and if applicable, details of automation tools used in the process. | 3-4 |
| Data items | 10a | List and define all outcomes for which data were sought. Specify whether all results that were compatible with each outcome domain in each study were sought (e.g. for all measures, time points, analyses), and if not, the methods used to decide which results to collect. | NA |
|  | 10b | List and define all other variables for which data were sought (e.g. participant and intervention characteristics, funding sources). Describe any assumptions made about any missing or unclear information. | 3-4 |
| Study risk of bias assessment | 11 | Specify the methods used to assess risk of bias in the included studies, including details of the tool(s) used, how many reviewers assessed each study and whether they worked independently, and if applicable, details of automation tools used in the process. | NA |
| Effect measures | 12 | Specify for each outcome the effect measure(s) (e.g. risk ratio, mean difference) used in the synthesis or presentation of results. | NA |
| Synthesis methods | 13a | Describe the processes used to decide which studies were eligible for each synthesis (e.g. tabulating the study intervention characteristics and comparing against the planned groups for each synthesis (item #5)). | 3-4 |
|  |  |  |  |
|  | 13b | Describe any methods required to prepare the data for presentation or synthesis, such as handling of missing summary statistics, or data conversions. | NA |
|  | 13c | Describe any methods used to tabulate or visually display results of individual studies and syntheses. | 3-4 |
|  | 13d | Describe any methods used to synthesize results and provide a rationale for the choice(s). If meta-analysis was performed, describe the model(s), method(s) to identify the presence and extent of statistical heterogeneity, and software package(s) used. | 3-4 |
|  | 13e | Describe any methods used to explore possible causes of heterogeneity among study results (e.g. subgroup analysis, meta-regression). | NA |
|  | 13f | Describe any sensitivity analyses conducted to assess robustness of the synthesized results. | NA |
| Reporting bias assessment | 14 | Describe any methods used to assess risk of bias due to missing results in a synthesis (arising from reporting biases). | NA |
| Certainty assessment | 15 | Describe any methods used to assess certainty (or confidence) in the body of evidence for an outcome. | NA |
| **RESULTS** | | |  |
| Study selection | 16a | Describe the results of the search and selection process, from the number of records identified in the search to the number of studies included in the review, ideally using a flow diagram. | 4-6 |
|  | 16b | Cite studies that might appear to meet the inclusion criteria, but which were excluded, and explain why they were excluded. | NA |
| Study characteristics | 17 | Cite each included study and present its characteristics. | Supplementary |
| Risk of bias in studies | 18 | Present assessments of risk of bias for each included study. | NA |
| Results of individual studies | 19 | For all outcomes, present, for each study: (a) summary statistics for each group (where appropriate) and (b) an effect estimate and its precision (e.g. confidence/credible interval), ideally using structured tables or plots. | NA |
| Results of syntheses | 20a | For each synthesis, briefly summarise the characteristics and risk of bias among contributing studies. | NA |
|  | 20b | Present results of all statistical syntheses conducted. If meta-analysis was done, present for each the summary estimate and its precision (e.g. confidence/credible interval) and measures of statistical heterogeneity. If comparing groups, describe the direction of the effect. | 4-10 |
|  | 20c | Present results of all investigations of possible causes of heterogeneity among study results. | NA |
|  | 20d | Present results of all sensitivity analyses conducted to assess the robustness of the synthesized results. | NA |
| Reporting biases | 21 | Present assessments of risk of bias due to missing results (arising from reporting biases) for each synthesis assessed. | NA |
| Certainty of evidence | 22 | Present assessments of certainty (or confidence) in the body of evidence for each outcome assessed. | NA |
| **DISCUSSION** | | |  |
| Discussion | 23a | Provide a general interpretation of the results in the context of other evidence. | 10-14 |
|  | 23b | Discuss any limitations of the evidence included in the review. | 13 |
|  | 23c | Discuss any limitations of the review processes used. | 13 |
|  | 23d | Discuss implications of the results for practice, policy, and future research. | 14 |
| **OTHER INFORMATION** | | |  |
| Registration and protocol | 24a | Provide registration information for the review, including register name and registration number, or state that the review was not registered. | 3-4 |
|  | 24b | Indicate where the review protocol can be accessed, or state that a protocol was not prepared. | 3-4 |
|  | 24c | Describe and explain any amendments to information provided at registration or in the protocol. | NA |
| Support | 25 | Describe sources of financial or non-financial support for the review, and the role of the funders or sponsors in the review. | 16 |
| Competing interests | 26 | Declare any competing interests of review authors. | 16 |
| Availability of data, code and other materials | 27 | Report which of the following are publicly available and where they can be found: template data collection forms; data extracted from included studies; data used for all analyses; analytic code; any other materials used in the review. | NA |

# REFERENCES

1. Bianchi S, Mosca A, Dalla Volta A, et al. Maintenance versus discontinuation of androgen deprivation therapy during continuous or intermittent docetaxel administration in castration-resistant prostate cancer patients: A multicentre, randomised Phase III study by the Piemonte Oncology Network. *Eur J Cancer* 2021; 155: 127-135. 20210806. DOI: 10.1016/j.ejca.2021.06.034.

2. Cinciripini PM, Green CE, Shete S, et al. Smoking Cessation After Initial Treatment Failure With Varenicline or Nicotine Replacement: A Randomized Clinical Trial. *Jama* 2024; 331: 1722-1731. DOI: 10.1001/jama.2024.4183.

3. Fatori D, de Bragança Pereira CA, Asbahr FR, et al. Adaptive treatment strategies for children and adolescents with Obsessive-Compulsive Disorder: A sequential multiple assignment randomized trial. *Journal of Anxiety Disorders* 2018; 58: 42-50. DOI: <https://doi.org/10.1016/j.janxdis.2018.07.002>.

4. Fortney JC, Heagerty PJ, Bauer AM, et al. Study to promote innovation in rural integrated telepsychiatry (SPIRIT): Rationale and design of a randomized comparative effectiveness trial of managing complex psychiatric disorders in rural primary care clinics. *Contemp Clin Trials* 2020; 90. Article. DOI: 10.1016/j.cct.2019.105873.

5. Friedman BW, Chen YT, Campbell C, et al. A sequential, multiple-assignment, randomized trial of analgesic strategies for acute musculoskeletal Pain. *Am J Emerg Med* 2024; 82: 15-20. 20240509. DOI: 10.1016/j.ajem.2024.05.005.

6. Fu SS, Rothman AJ, Vock DM, et al. Optimizing Longitudinal Tobacco Cessation Treatment in Lung Cancer Screening: A Sequential, Multiple Assignment, Randomized Trial. *JAMA netw* 2023; 6: E2329903. Article. DOI: 10.1001/jamanetworkopen.2023.29903.

7. Gao K, Arnold JG, Prihoda TJ, et al. Sequential Multiple Assignment Randomized Treatment (SMART) for Bipolar Disorder at Any Phase of Illness and at least Mild Symptom Severity. *Psychopharmacol Bull* 2020; 50: 8-25. Article.

8. Geng EH, Odeny TA, Montoya LM, et al. Adaptive Strategies for Retention in Care among Persons Living with HIV. *NEJM Evid* 2023; 2. Randomized Controlled Trial. DOI: <https://dx.doi.org/10.1056/evidoa2200076>.

9. Grilo CM, White MA, Masheb RM, et al. Randomized controlled trial testing the effectiveness of adaptive 'SMART' stepped-care treatment for adults with binge-eating disorder comorbid with obesity. *Am Psychol* 2020; 75: 204-218. DOI: 10.1037/amp0000534.

10. Hammel P, Huguet F, van Laethem JL, et al. Effect of Chemoradiotherapy vs Chemotherapy on Survival in Patients With Locally Advanced Pancreatic Cancer Controlled After 4 Months of Gemcitabine With or Without Erlotinib: The LAP07 Randomized Clinical Trial. *Jama* 2016; 315: 1844-1853. DOI: 10.1001/jama.2016.4324.

11. Karp JF, Zhang J, Wahed AS, et al. Improving Patient Reported Outcomes and Preventing Depression and Anxiety in Older Adults With Knee Osteoarthritis: Results of a Sequenced Multiple Assignment Randomized Trial (SMART) Study. *Am J Geriatr Psychiatry* 2019; 27: 1035-1045. 20190321. DOI: 10.1016/j.jagp.2019.03.011.

12. Kasari C, Kaiser A, Goods K, et al. Communication interventions for minimally verbal children with autism: a sequential multiple assignment randomized trial. *J Am Acad Child Adolesc Psychiatry* 2014; 53: 635-646. 20140312. DOI: 10.1016/j.jaac.2014.01.019.

13. Kim JS, Asher CA, Burkhauser M, et al. Using a Sequential Multiple Assignment Randomized Trial (SMART) to Develop an Adaptive K–2 Literacy Intervention With Personalized Print Texts and App-Based Digital Activities. *AERA Open* 2019; 5: 2332858419872701. DOI: 10.1177/2332858419872701.

14. Lebeau B, Chastang C, Allard P, et al. Six vs twelve cycles for complete responders to chemotherapy in small cell lung cancer: definitive results of a randomized clinical trial. The "Petites Cellules" Group. *Eur Respir J* 1992; 5: 286-290.

15. Matthay KK, Reynolds CP, Seeger RC, et al. Long-Term Results for Children With High-Risk Neuroblastoma Treated on a Randomized Trial of Myeloablative Therapy Followed by 13-cis-Retinoic Acid: A Children's Oncology Group Study. *Journal of Clinical Oncology* 2009; 27: 1007-1013. DOI: 10.1200/jco.2007.13.8925.

16. McKay JR, Drapkin ML, Van Horn DH, et al. Effect of patient choice in an adaptive sequential randomization trial of treatment for alcohol and cocaine dependence. *J Consult Clin Psychol* 2015; 83: 1021-1032. 20150727. DOI: 10.1037/a0039534.

17. Morgenstern J, Kuerbis A, Shao S, et al. An efficacy trial of adaptive interventions for alcohol use disorder. *J Subst Abuse Treat* 2021; 123. DOI: 10.1016/j.jsat.2020.108264.

18. Morin CM, Edinger JD, Beaulieu-Bonneau S, et al. Effectiveness of sequential psychological and medication therapies for insomnia disorder a randomized clinical trial. *JAMA Psychiatry* 2020; 77: 1107-1115. DOI: 10.1001/jamapsychiatry.2020.1767.

19. Naar-King S, Ellis DA, Idalski Carcone A, et al. Sequential Multiple Assignment Randomized Trial (SMART) to Construct Weight Loss Interventions for African American Adolescents. *J Clin Child Adolesc Psychol* 2016; 45: 428-441. 20150210. DOI: 10.1080/15374416.2014.971459.

20. Patrick ME, Lyden GR, Morrell N, et al. Main outcomes of M-bridge: A sequential multiple assignment randomized trial (SMART) for developing an adaptive preventive intervention for college drinking. *J Consult Clin Psychol* 2021; 89: 601-614. DOI: 10.1037/ccp0000663

10.1037/ccp0000663.supp (Supplemental).

21. Pelham WE, Jr., Fabiano GA, Waxmonsky JG, et al. Treatment Sequencing for Childhood ADHD: A Multiple-Randomization Study of Adaptive Medication and Behavioral Interventions. *J Clin Child Adolesc Psychol* 2016; 45: 396-415. 20160216. DOI: 10.1080/15374416.2015.1105138.

22. Petracci E, Scarpi E, Passardi A, et al. Effectiveness of bevacizumab in first- and second-line treatment for metastatic colorectal cancer: ITACa randomized trial. *Ther Adv Med Oncol* 2020; 12: 1758835920937427. 20200723. DOI: 10.1177/1758835920937427.

23. Petry NM, Alessi SM, Rash CJ, et al. A randomized trial of contingency management reinforcing attendance at treatment: Do duration and timing of reinforcement matter? *J Consult Clin Psychol* 2018; 86: 799-809. DOI: 10.1037/ccp0000330.

24. Powell BL, Moser B, Stock W, et al. Arsenic trioxide improves event-free and overall survival for adults with acute promyelocytic leukemia: North American Leukemia Intergroup Study C9710. *Blood* 2010; 116: 3751-3757. 20100812. DOI: 10.1182/blood-2010-02-269621.

25. Sinyor M, Schaffer A and Levitt A. The Sequenced Treatment Alternatives to Relieve Depression (STAR*D) Trial: A Review. *The Canadian Journal of Psychiatry* 2010; 55: 126-135. DOI: 10.1177/070674371005500303.

26. Schlam TR, Baker TB, Piper ME, et al. What to do after smoking relapse? A sequential multiple assignment randomized trial of chronic care smoking treatments. *Addiction* 2024; 119: 898-914. 20240128. DOI: 10.1111/add.16428.

27. Schmitz JM, Stotts AL, Vujanovic AA, et al. Contingency management plus acceptance and commitment therapy for initial cocaine abstinence: Results of a sequential multiple assignment randomized trial (SMART). *Drug Alcohol Depend* 2024; 256: 111078. 20240103. DOI: 10.1016/j.drugalcdep.2023.111078.

28. Sherwood NE, Crain AL, Seburg EM, et al. BestFIT Sequential Multiple Assignment Randomized Trial Results: A SMART Approach to Developing Individualized Weight Loss Treatment Sequences. *Ann Behav Med* 2022; 56: 291-304. Article. DOI: 10.1093/abm/kaab061.

29. Sikorskii A, Badger T, Segrin C, et al. A Sequential Multiple Assignment Randomized Trial of Symptom Management After Chemotherapy. *J Pain Symptom Manage* 2023; 65: 541-552.e542. Article. DOI: 10.1016/j.jpainsymman.2023.02.005.

30. Smith SN, Almirall D, Choi SY, et al. Primary aim results of a clustered SMART for developing a school-level, adaptive implementation strategy to support CBT delivery at high schools in Michigan. *Implement Sci* 2022; 17. Article. DOI: 10.1186/s13012-022-01211-w.

31. Somers TJ, Winger JG, Fisher HM, et al. Behavioral cancer pain intervention dosing: Results of a Sequential Multiple Assignment Randomized Trial. *Pain* 2023; 164: 1935-1941. Article. DOI: 10.1097/j.pain.0000000000002915.

32. Swartz MS, Stroup TS, McEvoy JP, et al. What CATIE found: results from the schizophrenia trial. *Psychiatr Serv* 2008; 59: 500-506. DOI: 10.1176/ps.2008.59.5.500.

33. Thall PF, Logothetis C, Pagliaro LC, et al. Adaptive therapy for androgen-independent prostate cancer: a randomized selection trial of four regimens. *J Natl Cancer Inst* 2007; 99: 1613-1622. 20071030. DOI: 10.1093/jnci/djm189.

34. Tummarello D, Mari D, Graziano F, et al. A randomized, controlled phase III study of cyclophosphamide, doxorubicin, and vincristine with etoposide (CAV-E) or teniposide (CAV-T), followed by recombinant interferon-alpha maintenance therapy or observation, in small cell lung carcinoma patients with complete responses. *Cancer* 1997; 80: 2222-2229.

35. Wyatt G, Lehto R, Guha-Niyogi P, et al. Reflexology and meditative practices for symptom management among people with cancer: Results from a sequential multiple assignment randomized trial. *Res Nurs Health* 2021; 44: 796-810. Article. DOI: 10.1002/nur.22169.

36. Abuogi LL, Kulzer JL, Akama E, et al. Adapt for Adolescents: Protocol for a sequential multiple assignment randomized trial to improve retention and viral suppression among adolescents and young adults living with HIV in Kenya. *Contemp Clin Trials* 2023; 127: 107123. Clinical Trial Protocol

Research Support, Non-U.S. Gov't. DOI: <https://dx.doi.org/10.1016/j.cct.2023.107123>.

37. Arean P, Hull D, Pullmann MD and Heagerty PJ. Protocol for a sequential, multiple assignment, randomised trial to test the effectiveness of message-based psychotherapy for depression compared with telepsychotherapy. *BMJ Open* 2021; 11: e046958. Clinical Trial Protocol

Research Support, N.I.H., Extramural. DOI: <https://dx.doi.org/10.1136/bmjopen-2020-046958>.

38. Auyeung SF, Long Q, Royster EB, et al. Sequential multiple-assignment randomized trial design of neurobehavioral treatment for patients with metastatic malignant melanoma undergoing high-dose interferon-alpha therapy. *Clin* 2009; 6: 480-490.

39. Bahraini NH, Matarazzo BB, Barry CN, et al. Protocol: examining the effectiveness of an adaptive implementation intervention to improve uptake of the VA suicide risk identification strategy: a sequential multiple assignment randomized trial. *Implement Sci* 2020; 15: 58. Clinical Trial Protocol

Research Support, U.S. Gov't, Non-P.H.S. DOI: <https://dx.doi.org/10.1186/s13012-020-01019-6>.

40. Becker K, Banaschewski T, Brandeis D, et al. Individualised stepwise adaptive treatment for 3-6-year-old preschool children impaired by attention-deficit/hyperactivity disorder (ESCApreschool): study protocol of an adaptive intervention study including two randomised controlled trials within the consortium ESCAlife. *Trials* 2020; 21. Article. DOI: 10.1186/s13063-019-3872-8.

41. Belzer ME, MacDonell KK, Ghosh S, et al. Adaptive antiretroviral therapy adherence interventions for youth living with HIV through text message and cell phone support with and without incentives: protocol for a sequential multiple assignment randomized trial (SMART). *JMIR Res Protoc* 2018; 7: e11183.

42. Buchholz SW, Wilbur J, Halloway S, et al. Study protocol for a sequential multiple assignment randomized trial (SMART) to improve physical activity in employed women. *Contemp Clin Trials* 2020; 89: 105921.

43. Carr E, Whiston A, O'Reilly S, et al. Sequential multiple assignment randomised trial to develop an adaptive mobile health intervention to increase physical activity in people poststroke in the community setting in Ireland: TAPAS trial protocol. *BMJ Open* 2024; 14: e072811. 20240118. DOI: 10.1136/bmjopen-2023-072811.

44. Comins CA, Schwartz SR, Phetlhu DR, et al. Siyaphambili protocol: an evaluation of randomized, nurse‐led adaptive HIV treatment interventions for cisgender female sex workers living with HIV in Durban, South Africa. *Res Nurs Health* 2019; 42: 107-118.

45. Drake CL, Kalmbach DA, Cheng P, et al. Sleep to Reduce Incident Depression Effectively (STRIDE): study protocol for a randomized controlled trial comparing stepped-care cognitive-behavioral therapy for insomnia versus sleep education control to prevent major depression. *Trials* 2022; 23. Article. DOI: 10.1186/s13063-022-06850-4.

46. Edelman EJ, Dziura J, Deng Y, et al. A SMARTTT approach to Treating Tobacco use disorder in persons with HIV (SMARTTT): Rationale and design for a hybrid type 1 effectiveness-implementation study. *Contemp Clin Trials* 2021; 110. Article. DOI: 10.1016/j.cct.2021.106379.

47. Eldridge-Smith ED, Manber R, Tsai S, et al. Stepped care management of insomnia co-occurring with sleep apnea: the AIR study protocol. *Trials* 2022; 23. Article. DOI: 10.1186/s13063-022-06753-4.

48. Fernandez ME, Schlechter CR, Del Fiol G, et al. QuitSMART Utah: an implementation study protocol for a cluster-randomized, multi-level sequential multiple assignment randomized trial to increase reach and impact of tobacco cessation treatment in community health centers. *Implement Sci* 2020; 15: 1-13.

49. Flynn D, Eaton LH, Langford DJ, et al. A SMART design to determine the optimal treatment of chronic pain among military personnel. *Contemp Clin Trials* 2018; 73: 68-74.

50. Fox CK, Vock DM, Sherwood NE, et al. SMART use of medications for the treatment of adolescent severe obesity: A sequential multiple assignment randomized trial protocol. *Contemp Clin Trials* 2024: 107444. DOI: <https://dx.doi.org/10.1016/j.cct.2024.107444>.

51. Fritz JM, Rhon DI, Teyhen DS, et al. A Sequential Multiple-Assignment Randomized Trial (SMART) for stepped care management of low back pain in the military health system: A trial protocol. *Pain Med* 2020; 21: S73-S82. DOI: 10.1093/pm/pnaa338.

52. Germeroth LJ, Benno MT, Conlon RPK, et al. Trial design and methodology for a non-restricted sequential multiple assignment randomized trial to evaluate combinations of perinatal interventions to optimize women's health. *Contemp Clin Trials* 2019; 79: 111-121.

53. Hassett AL, Williams DA, Harris RE, et al. An Interventional Response Phenotyping Study in Chronic Low Back Pain: Protocol for a Mechanistic Randomized Controlled Trial. *Pain Med* 2023; 24: S126-S138. Article. DOI: 10.1093/pm/pnad005.

54. van Heerden A, Szpiro A, Ntinga X, et al. A Sequential Multiple Assignment Randomized Trial of scalable interventions for ART delivery in South Africa: the SMART ART study. *Trials* 2023; 24. Article. DOI: 10.1186/s13063-022-07025-x.

55. Hibbard JC, Friedstat JS, Thomas SM, et al. LIBERTI: A SMART study in plastic surgery. *Clin* 2018; 15: 286-293.

56. Johnson JE, Wiltsey-Stirman S, Sikorskii A, et al. Protocol for the ROSE sustainment (ROSES) study, a sequential multiple assignment randomized trial to determine the minimum necessary intervention to maintain a postpartum depression prevention program in prenatal clinics serving low-income women. *Implement Sci* 2018; 13: 1-12.

57. Kilbourne AM, Almirall D, Eisenberg D, et al. Protocol: Adaptive Implementation of Effective Programs Trial (ADEPT): cluster randomized SMART trial comparing a standard versus enhanced implementation strategy to improve outcomes of a mood disorders program. *Implement Sci* 2014; 9: 1-14.

58. Kopelowicz A, Nandy K, Ruiz ME, et al. Improving Self-management of Type 2 Diabetes in Latinx Patients: Protocol for a Sequential Multiple Assignment Randomized Trial Involving Community Health Workers, Registered Nurses, and Family Members. *JMIR Res Protoc* 2023; 12. Article. DOI: 10.2196/44793.

59. Kor PPK, Chou KL, Zarit SH, et al. Sequential multiple assignment randomised controlled trial protocol for developing an adaptive intervention to improve depressive symptoms among family caregivers of people with dementia. *BMJ Open* 2023; 13. Article. DOI: 10.1136/bmjopen-2023-072410.

60. Levy R, Mathai M, Chatterjee P, et al. Implementation research for public sector mental health care scale-up (SMART-DAPPER): a sequential multiple, assignment randomized trial (SMART) of non-specialist-delivered psychotherapy and/or medication for major depressive disorder and posttraumatic stress disorder (DAPPER) integrated with outpatient care clinics at a county hospital in Kenya. *BMC Psychiatry* 2019; 19: 1-14.

61. Li X, Guo X, Fan X, et al. Sequential Multiple-Assignment Randomized Trials to Compare Antipsychotic Treatments (SMART-CAT) in first-episode schizophrenia patients: Rationale and trial design. *Schizophr Res* 2021; 230: 87-94. DOI: 10.1016/j.schres.2020.11.010.

62. Liu H, Chen G, Li J, et al. Sequential multiple assignment randomised trial of a brief contact intervention for suicide risk management among discharged psychiatric patients: An implementation study protocol. *BMJ Open* 2021; 11. Review. DOI: 10.1136/bmjopen-2021-054131.

63. Mustanski B, Moskowitz DA, Moran KO, et al. Evaluation of a stepped-care eHealth HIV prevention program for diverse adolescent men who have sex with men: protocol for a hybrid type 1 effectiveness implementation trial of SMART. *JMIR Res Protoc* 2020; 9. Article. DOI: 10.2196/19701.

64. Nelson B, Amminger GP, Yuen HP, et al. Staged treatment in early psychosis: a sequential multiple assignment randomised trial of interventions for ultra high risk of psychosis patients. *Early Interv Psychiatry* 2018; 12: 292-306.

65. O’Keefe VM, Haroz EE, Goklish N, et al. Employing a sequential multiple assignment randomized trial (SMART) to evaluate the impact of brief risk and protective factor prevention interventions for American Indian Youth Suicide. *BMC Public Health* 2019; 19: 1-12.

66. Osilla KC, Gore KL, Pedersen ER, et al. Study protocol for a sequential multiple assignment randomized trial to reduce risky drinking among service members and their partners. *Contemp Clin Trials* 2023; 133. Article. DOI: 10.1016/j.cct.2023.107324.

67. Peter SC, Murphy JG, Witkiewitz K, et al. Use of a sequential multiple assignment randomized trial to test contingency management and an integrated behavioral economic and mindfulness intervention for buprenorphine-naloxone medication adherence for opioid use disorder. *Trials* 2023; 24. Article. DOI: 10.1186/s13063-023-07102-9.

68. Peterson BS, West AE, Weisz JR, et al. A sequential Multiple Assignment Randomized Trial (SMART) study of medication and CBT sequencing in the treatment of pediatric anxiety disorders. *BMC Psychiatry* 2021; 21. DOI: 10.1186/s12888-021-03314-y.

69. Quanbeck A, Almirall D, Jacobson N, et al. The Balanced Opioid Initiative: Protocol for a clustered, sequential, multiple-assignment randomized trial to construct an adaptive implementation strategy to improve guideline-concordant opioid prescribing in primary care. *Implement Sci* 2020; 15. Article. DOI: 10.1186/s13012-020-00990-4.

70. Rabin BA, Cain KL, Watson P, Jr., et al. Scaling and sustaining COVID-19 vaccination through meaningful community engagement and care coordination for underserved communities: hybrid type 3 effectiveness-implementation sequential multiple assignment randomized trial. *Implement Sci* 2023; 18: 28. Randomized Controlled Trial. DOI: <https://dx.doi.org/10.1186/s13012-023-01283-2>.

71. Sabri B, Glass N, Murray S, et al. A technology-based intervention to improve safety, mental health and empowerment outcomes for immigrant women with intimate partner violence experiences: it’s weWomen plus sequential multiple assignment randomized trial (SMART) protocol. *BMC Public Health* 2021; 21. Article. DOI: 10.1186/s12889-021-11930-2.

72. Smith SK, Somers TJ, Kuhn E, et al. A SMART approach to optimizing delivery of an mHealth intervention among cancer survivors with posttraumatic stress symptoms. *Contemp Clin Trials* 2021; 110. Article. DOI: 10.1016/j.cct.2021.106569.

73. Sripada RK, Smith K, Walters HM, et al. Testing adaptive interventions to improve PTSD treatment outcomes in Federally Qualified Health Centers: Protocol for a randomized clinical trial. *Contemp Clin Trials* 2023; 129. Article. DOI: 10.1016/j.cct.2023.107182.

74. Velloza J, Poovan N, Ndlovu N, et al. Adaptive HIV pre-exposure prophylaxis adherence interventions for young South African women: Study protocol for a sequential multiple assignment randomized trial. *PLoS ONE* 2022; 17. Article. DOI: 10.1371/journal.pone.0266665.

75. Walton MA, Carter PM, Seewald L, et al. Adaptive interventions for alcohol misuse and violent behaviors among adolescents and emerging adults in the emergency department: A sequential multiple assignment randomized controlled trial protocol. *Contemp Clin Trials* 2023; 130. Article. DOI: 10.1016/j.cct.2023.107218.

76. Wan Y, Cai Y, Liao S, et al. Smartphone-based versus traditional face-to-face collaborative care for community-dwelling older adults living with dementia in China: protocol for an implementation science-based sequential multiple assignment randomised trial. *BMJ Open* 2023; 13. Article. DOI: 10.1136/bmjopen-2022-067406.

77. Windsor L, Benoit E, R MP and Sarol J. Optimization of a new adaptive intervention using the SMART Design to increase COVID-19 testing among people at high risk in an urban community. *Trials [Electronic Resource]* 2022; 23: 310. Randomized Controlled Trial. DOI: <https://dx.doi.org/10.1186/s13063-022-06216-w>.

78. Zhao SZ, Weng X, Luk TT, et al. Adaptive interventions to optimise the mobile phone-based smoking cessation support: study protocol for a sequential, multiple assignment, randomised trial (SMART). *Trials* 2022; 23. Article. DOI: 10.1186/s13063-022-06502-7.

79. Zhou G, Lee M-C, Atieli HE, et al. Adaptive interventions for optimizing malaria control: an implementation study protocol for a block-cluster randomized, sequential multiple assignment trial. *Trials* 2020; 21. Article. DOI: 10.1186/s13063-020-04573-y.

80. Zullig LL, Shahsahebi M, Neely B, et al. Low-touch, team-based care for co-morbidity management in cancer patients: the ONE TEAM randomized controlled trial. *BMC Fam Pract* 2021; 22. Article. DOI: 10.1186/s12875-021-01569-8.
